# Epidemiological and clinical characteristics of children who died from hand, foot and mouth disease in Vietnam, 2011

**DOI:** 10.1186/1471-2334-14-341

**Published:** 2014-06-18

**Authors:** Ngoc TB Nguyen, Hau V Pham, Cuong Q Hoang, Tien M Nguyen, Long T Nguyen, Hung C Phan, Lan T Phan, Long N Vu, Nguyen N Tran Minh

**Affiliations:** 1 Department of Epidemiology, Institute of Hygiene and Public Health in Ho Chi Minh City, 159 Hung Phu Street, District 8, Ho Chi Minh City, Vietnam; 2 Vietnam Field Epidemiology Training Program, Room 407, A6 Building, 1 Ton That Tung Street, Dong Da District, Ha Noi, Vietnam; 3 Pasteur Institute in Ho Chi Minh City, 167 Pasteur Street, 8 Ward, 3 District, Ho Chi Minh City, Vietnam; 4 Children Hospital, 1, 341 Su Van Hanh Street, District 8, Ho Chi Minh City, Vietnam; 5 Vietnam Ministry of Health, 138A Giang Vo Street, Ba Dinh District, Ha Noi, Vietnam

**Keywords:** Hand foot and mouth disease, Clinical manifestation, Enterovirus, Vietnam

## Abstract

**Background:**

In 2011, a large outbreak of hand, foot and mouth disease (HFMD) in Vietnam resulted in 113,121 children seeking medical attention, of whom170 died. Understanding the epidemiology of fatal HFMD may improve treatment and help targeting prevention activities for vulnerable populations. We describe epidemiological and clinical characteristics of children who died from HFMD in Vietnam in 2011.

**Methods:**

Clinical data were obtained through reviewing medical records of the deaths occurring from January through December 2011 in all hospitals in Vietnam. Hospitals reported any deaths among patients with laboratory-confirmed enterovirus (EV) infection to the Ministry of Health. Data were extracted from the national database.

**Results:**

Of the 169 deaths reviewed for whom records were available, 87% were 3-year-old or younger, 69% were male, 18% attended daycare, 89% lived in Southern Vietnam, and 85% of the deaths occurred between May-October 2011. One hundred thirty (77%) cases sought treatment in a hospital within three days of onset of illness. Symptoms at admission included fever (98%), myoclonus (66%), vomiting (53%), oral ulcers (50%) and vesicular erythema (50%). One hundred six (75%) cases had leukocytosis and 91 (54%) had hyperglycemia. One hundred three (61%) tested positive for EV, of which 84 (82%) were positive for EV71.

**Conclusions:**

Deaths associated with HFMD occurred throughout 2011 among males three years or younger who were cared for at home. The HFMD control program should focus on interventions at the household level. Clinicians should be alerted to symptoms suggestive of severe HFMD including fever, myoclonus, vomiting, oral ulcers and vesicles with high white blood cell count especially in young children.

## Background

Hand, foot and mouth disease (HFMD) is a common infectious disease caused by a group of enteroviruses, most frequently Coxsackie A 16 (CAV16) and Enterovirus 71 (EV71) [1,2]. Typical clinical manifestations of HFMD in children include fever, skin eruptions on hands and feet, and vesicles in the mouth. However, cases involving the central nervous system and/or pulmonary edema have also been reported [3]. There are no vaccines neither specific treatment for this disease.

Alsop et al. introduced the term HFMD when they described an outbreak that occurred in the summer of 1959 in Birmingham [4]. The disease then gradually spread around the world [5,6]. In 2009, an outbreak in the mainland China involved 1,155,525 cases, 13,810 severe cases and 353 deaths. Outbreaks have been reported in other countries in the Western Pacific Region, including Australia, Brunei Darussalam, Japan, Malaysia, Mongolia, the Republic of Korea, Singapore, and Vietnam [5].

In Vietnam, the first case of HFMD was reported in 2003 [5] and within the following years the disease was reported in all major cities and provinces in the country. The number of reported cases and deaths of HFMD were 5719 and 23 in 2007; 10,958 and 25 in 2008; and 10632 and 23 in 2009, respectively [5]. National surveillance data obtained by the Ministry of Health showed that there has been an increasing trend in recent years which peaked in 2011 when Vietnam recorded 113,121 cases of HFMD and 170 deaths [7]. Since 2011, the Ministry of Health classified HFMD as a severe infectious disease with outbreak potential (class B communicable disease) and the disease has been reported weekly by the national communicable disease surveillance system which collects reports from all the hospitals.

Little is known about epidemiology of HFMD in the Vietnamese population. In 2005, a sentinel surveillance system at a pediatric hospital diagnosed 764 children with HFMD in Ho Chi Minh City [8]. Among them, 96% were five years of age or younger. All cases had specimens taken for virological investigation. Human EV was isolated from 411 (54%) patients. Of those, 173 (42%) were identified as EV71 and 214 (52%) as CAV16. Of the patients with EV71 infection, 51 (29%) had severe neurological complications and three were fatal. In 2006–2007, sentinel surveillance at the same hospital reported 305 cases diagnosed with a neurological disease, of which 36 cases (11%) and three deaths were associated with EV71 [5].

To reduce severe illness and death from HFMD in Vietnam, public health practitioners and clinicians need more comprehensive epidemiological intelligence to better target prevention and control measures, and provide case management. The present study describes epidemiological and clinical characteristics of patients who died from HFMD in hospitals in Vietnam in 2011.

## Methods

A descriptive study was conducted from May to November, 2012 by extracting, compiling and reviewing the medical records of all patients who died from HFMD in hospitals in Vietnam in 2011.

Data collected on subjects included, age, gender, clinical status, use of medication, results of laboratory tests, clinical grade at hospital admission, treatment, date of diagnosis, and date of death. A standard form (see Additional file 1) was used to abstract these data from medical records. Study clinicians reviewed all data entered from medical records for accuracy and completeness.

In Vietnam, HFMD is defined as a brief febrile illness in children accompanied by typical skin rash, with or without mouth ulcers. The rash is papulo-vesicular occurring on the palms or soles of the feet, or both. In young children or infants the rash may be maculo-papular without vesicles and may also involve the buttocks, knees or elbows [9]. A confirmed HFMD case was defined as a patient who had a positive RT–PCR assay for EV71 or other EV [10]. Since the beginning of 2011, hospitals reported every HFMD death (with or without laboratory confirmation). From July 2011, as laboratory testing capacity became more widely available, hospitals were requested to report fatal cases of HFMD with laboratory confirmation of EV infection [11].

The Vietnam Ministry of Health established the following grading for HFMD at admission: Grade 1 is uncomplicated disease with fever and vesicles or papules on hands, feet, buttocks, and oral mucosa; Grade 2a is presence of myoclonus as reported by the caregiver; Grade 2b is when myoclonus is observed by a physician; Grade 3 is presence of autonomic dysfunction with fever that is unresponsive to antipyretics, hypertension and persistent tachycardia; Grade 4 exhibits cardiopulmonary compromise with pulmonary edema or hemorrhage. Grades 2b, 3, and 4 describe severe disease and are indicators for hospital admission and treatment.

The researchers were trained on the protocol and the procedure of quality assurance. All of the completed questionnaires were examined and corrected before entering into the EpiData3.02 database. Subsequently, the supervisor checked and corrected inconsistent data to assure the quality of the dataset. Each patient record was assigned a unique identification number. Statistical analysis was done by EpiData. Descriptive statistics, including means for the continuous variables and proportions for the categorical variables were done to describe patient characteristics.

Ethical clearance was not required as the study was part of an emergency response of the Vietnam Ministry of Health.

## Results

**Figure 1:**
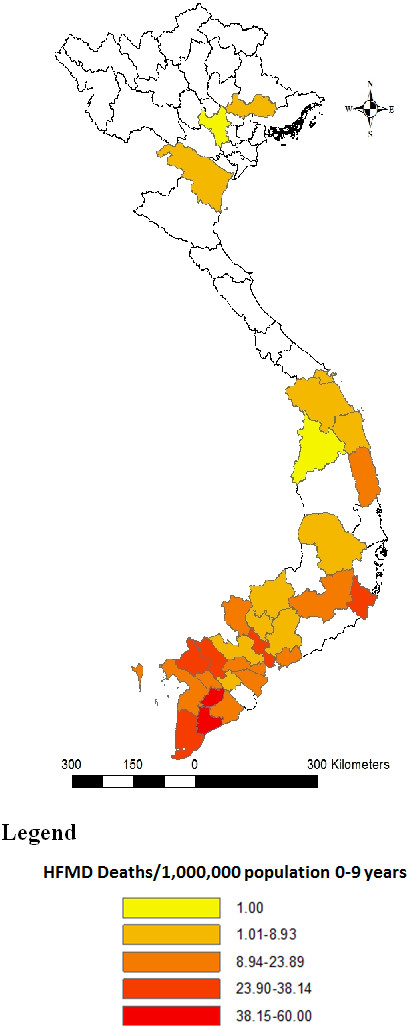
Distribution of deaths caused by Hand Foot and Mouth Disease per 1,000,000 population (aged 0–9 years) by provinces in Vietnam 2011.

**Figure 2:**
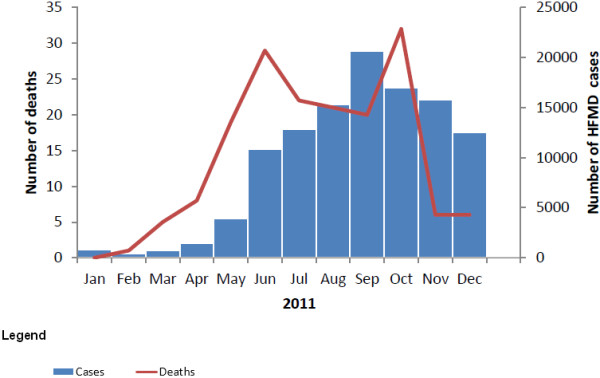
Distribution of cases and deaths caused by hand foot and mouth disease by month of onset in Vietnam 2011.

Of the 170 fatal cases reported, 169 (99%) had medical records available for review. Case fatality was 1.5 per 1,000 reported cases. Deaths due to HFMD occurred in all regions of Vietnam, from Northern to Central and Southern. However, the majority of HFMD cases and deaths were in the Southern provinces (Figure 1). Place of death by regional, provincial and district hospital was 139/169 (82%), 28/169 (17%) and 2/169 (1%), respectively. HFMD deaths occurred virtually throughout the year, with 85% occurring from May to October in 2011. Peaks occurred in June and October of which October peak was slightly higher than the June peak (Figure 2).

The demographic characteristics showed that 87% (147/169) of HFMD deaths occurred in children aged 3 years or younger and 69% (116/169) were boys; the male-to-female ratio was 2.2 per 1. Only 18% (10/56) of the cases had history of daycare attendance and 31% (10/32) had known contact with HFMD cases prior to illness. Seventy seven percent of the cases (130/169) sought treatment in hospital within three days of illness onset, and 61% (62/102) were self-admitted in severe condition. Two cases (3%) were dead upon arrival in the hospital (Table 1).

**Table 1:** Epidemiological characteristics of deaths caused by hand foot and mouth disease in Vietnam 2011

**Characteristic**	**No. of cases/total (%)**
**Demographic**	
Male sex	116/169 (69)
Age in month, Median (range)	25 (3–107)
Age group ≤ 3 years	147/169 (87)
**HFMD exposure**	
Daycare attendance	10/56 (18)
Known contact with HFMD cases	10/32 (31)
**Disease recognition, referral and progression**	
Admission within 3 days of symptom recognition	130/169 (77)
Self-admission in severe state	62/102 (61)
Fatal cases before admission	2/67 (3)
Transferred to provincial or tertiary level hospital	54/166 (33)
Diagnosis of HFMD at referral	29/44 (66)
Referral under critical conditions	26/67 (39)
Referral cases died before admission	4/67 (6)
Days from referral hospital admission to death, median (inter-quartile range)	1 (1–3)
**Fatal cases at hospital levels**	
Tertiary level	139/169 (82)
Province level	28/169 (17)
District level	2/169 (1)
**Time from admission to death, median (range)**	
Tertiary level	1 (0–33)
Province level	1 (0–7)
District level	0.5 (0–1)

Thirty three percent (54/166) was transferred to higher level hospitals; 66% (29/44) of the cases had HFMD diagnosed at referral; 39% (26/67) were referred under critical conditions, in which 6% cases (4/67) were dead on arrival. Median time from admission to a referral hospital to death was one day (Table 1).

Symptoms recorded at hospital admission included fever 98% (165/169), myoclonus 66% (87/131), vomiting 53% (70/131), oral ulcers 50% (60/119), vesicular erythema 50% (62/125), and diarrhea 10% (11/114).

Ninety three percent (158/169) of the cases had a HFMD grading at the time of admission. Of these, 90% (142/158) had HFMD grade 2a, 2b, 3 or 4 (Table 2). Median (range) time from the onset of illness to admission of grade 1, 2a, 2b, 3 or 4 and misdiagnosed patients were not statistically different: 2.5 (1–4), 3 (1–6), 2 (1–6), 3 (1–21) and 3 (2–6) days, respectively. Median (range) time from the admission to death of grade 1, 2a, 2b, 3 or 4 reduced gradually from 2 (1–7), 2 (0–46), 2 (0–14), 1 (0–33) days, respectively, data on misdiagnosed patients was missing.

**Table 2:** Clinical characteristics of deaths caused by hand foot and mouth disease in Vietnam 2011

**Characteristic**	**No. of cases/total (%)**
**Symptoms recorded at admission in terminal hospital**	
Fever	165/169 (98)
Myoclonus	87/131 (66)
Vomiting	70/131 (53)
Oral ulcers	60/119 (50)
Vesicular erythema	62/125 (50)
Diarrhea	11/114 (10)
Shock	74/169 (44)
Respiratory failure/cyanosis	69/169 (41)
Pulmonary edema	37/169 (22)
Apnea /Dyspnea	14/169 (8)
**Highest clinical grade recorded at admission in terminal hospital**	
1	10/158 (6)
2a	53/158 (34)
2b	26/158 (17)
3 or 4	63/158 (40)
Misdiagnosis	6/158 (4)
**Laboratory results during hospitalization**	
White blood cell count > 16000/mm^3^*	106/142 (75)
Blood sugar > 180 mg/dL*	76/142 (54)
Severe Metabolic acidosis	69/140 (49)
Troponin I (+)	63/142 (44)
Platelet > 400,000/mm^3^*	57/142 (40)
**EV test results**	
EV71	84/103 (82)
EV	16/103 (16)
**Main causes of death**	
Respiratory failure	123/150 (82)
Prolonged shock	104/150 (69)
Coma	83/150 (55)
Ventricular fibrillation	11/150 (7)
Others (heart failure, cerebral edema ect.)	17/150 (11)
**Treatment**	
Vasoactive drugs	
Dobutamine	153/169 (91)
Adrenaline	102/169 (60)
Noradrenaline	51/169 (30)
Dopamine	26/169 (15)
Intravenous immunoglobulin	159/169 (94)
Hemofiltration	35/169 (21)

*Normal range:

- White blood cell count (4,300-10,800/mm^3^).

- Blood sugar (70–130 mg/dl).

- Platelet (100,000-300,000/mm^3^).

Laboratory results during hospitalization showed that 75% (106/142) of the cases had an elevated white blood cell count greater than 16000/mm^3^, 40% (57/142) had a platelet count greater than 400,000/mm^3^, 53% (76/142) had blood sugar level greater than 180 mg/dL, 49% (69/140) had severe metabolic acidosis, and 44% (63/142) had Troponin I (+). Most (82%) of the cases tested positive for EV71 (Table 2).

The severe cases were treated with vasoactive drugs. Ninety one percent (153/169) of the cases were given dobutamine, 60% (102/169) adrenalin, 30% (51/169) noradrenaline, and 15% (26/169) dopamine. Respiratory failure was responsible for 82% (123/150) of the deaths, prolonged shock for 69% (104/150), and coma for 55% (83/150) (Table 2).

Ninety four percent (159/169) of the cases received intravenous immunoglobulin. Hemofiltration was performed on 21% (35/169) of the fatal cases.

## Discussion

A large outbreak of HFMD occurred in Vietnam in 2011. The death of 170 children from HFMD allowed the opportunity to describe and better understand how so many young children died.

The median age of children who died from HFMD in this study was higher than those found in Taiwan and Malaysia (25 months vs. 17 months and 18 months, respectively) [12,13], but it was the same in Singapore in 2000 [6]. However, the proportion of cases aged 3 years or younger were the same in Taiwan (79% vs. 78%) [12]. The ratio of male and female was 2.2:1, higher than in outbreaks in other countries such as 1.4:1 in Taiwan [12], 1.3:1 in Singapore [6] and 1.9:1 in Malaysia [13]. We found no plausible explanation for this skewed ratio.

The exposure history of the cases, i.e. those who attended daycare or had known contact with other HFMD cases, was poorly recorded and did not indicate any particular source of transmission. HFMD is known to spread through direct contact with mucus, saliva, or feces of an infected person. Data from this study showed that only 18% of the fatal cases attended daycare which suggested that most of the HFMD transmission occurred at home and could be from contact with other family members having asymptomatic or mild infection such as parents, older siblings, nannies and other caregivers [14]. Therefore, health education efforts including behavior change communication to prevent HFMD transmission should be conducted not only in school but widely in the community, targeting households and families with young children. Further studies on virus circulation and virulence in different populations and settings are needed to provide a rational basis for targeting prevention and control measures.

Most of HFMD cases and deaths were reported in Southern provinces, from May to October. The occurrence of HFMD during the rainy season (May to October) was higher than the dry season (November to April): 90% vs. 10%. The mean air temperature of Southern provinces was always higher than other provinces (mean air 27.5°C in southern provinces (Ca Mau) compared with range 18.1-26.9°C in other provinces). The monthly air temperature from March to November was higher than December, January and February (range 27.2-28.7 vs. 26.3-26.4°C) [15].

There were two peaks of HFMD deaths. In October, at the peak of the rainy season, HFMD deaths reached the highest number as the epidemic spread to the North where health workers had limited experience in case management of HFMD. A study in Hong Kong on the relationship between meteorological parameters and HFMD activity showed that meteorological parameters helped in predicting HFMD activity and could assist in explaining the winter peak detected in recent years and in issuing early warning [16]. Other studies on the association between meteorological parameters and occurrence of HFMD are warranted. In Vietnam, HFMD surveillance data need to be compiled for some more years to demonstrate the seasonality as HFMD is a newly emerging disease.

Fever was reported in most cases, followed by myoclonus which was markedly higher than other symptoms. In reality, the myoclonus symptoms could be observed more frequently than in this study [17]. In a survey at Children Hospital Number 2 in Ho Chi Minh City, myoclonus was observed in almost all (98%) severe cases [18]. The explanation for the low rate of myoclonus could be due to missing data in medical records. Proportions of the cases having fever and oral ulcer were slightly lower than in a study in Sarawak, Malaysia (fever 100%, oral ulcer 66%) [13] and another study in Peninsular Malaysia in 1997 (fever 100%, oral ulcer 72%) [19]. Taken together, warning signs of severe HFMD could be considered as high fever, myoclonus and persistent vomiting with or without oral ulcers and vesicular erythema.

The classification of HFMD in Vietnam [9] is different from the WHO guide [5], however the Vietnamese clinical grading is generally consistent with that of WHO. Almost all HFMD fatalities were from grade 2a or above at admission. Given the rapid clinical progression of HFMD, clinicians should be able to recognize early the uncomplicated forms of the disease (grades 1 and 2a) as their proportions were found higher in the present study than those found in a previous study in Vietnam [20]. It is also important to note that 34% of HFMD were misdiagnosed even upon referral to provincial or regional hospitals. Although clinicians working at the provincial and district hospitals have received training on case management of HFMD according to the Ministry of Health guidelines [11], it was uncertain whether they could apply the guidelines at their respective level of care.

Laboratory abnormalities of the cases included elevated levels of white blood cell count, blood sugar, severe metabolic acidosis, Troponin I, and platelet which may help to predict a poor outcome [19,21]. A study to determine the risk factors predictive of death from HFMD in Singapore showed that elevated white blood cell count was a risk factor and should alert the physician of a fatal course of illness [6,22].

There are no specific treatments for HFMD. In this study, dobutamine was used in almost all (96%) cases as recommended by WHO [5] and the Ministry of Health [9]. On the contrary, dopamine which increases sympathomimetics, production of cytokines, inflammation and severity of disease was administered to 18% of cases. Dopamine has been considered as one of potential risk factors contributing to fatal outcome of HFMD [5]. Respiratory failure, prolonged shock and coma were recorded as main causes of deaths. Similar results were found in studies conducted in Taiwan [12] and Malaysia [19]. The rapid onset and progression of pulmonary and cardiac failure in previously healthy children stand out as a unique feature of this disease [13,17]. As the median time from referral hospital admission to death was only 1 day, only few patients had access to hemofiltration which is considered to have some therapeutic effects on HFMD. In view of this, more timely referral or initiating hemofiltration treatment prior to referral at the provincial hospitals may potentially be lifesaving.

This is the first national study describing a large number of deaths caused by HFMD during an outbreak in Vietnam in 2011. In this respect it has the requisites to provide an overall epidemiological picture and reveal some factors potentially associated with the deaths. However, some limitations of this study merit noting. Firstly, the retrospective study could not obtain key epidemiological and clinical data of HFMD patients during the time of disease. Secondly, patient records may have been incompletely filled. Despite these limitations, the results provided some hypothesis for further studies. An analytical study may help to identify risk factors of acquiring severe HFMD that can potentially be prevented. Understanding these factors will provide a rational basis for developing successful preventive interventions and treatments of severe HFMD.

## Conclusions

Clinicians should be alerted to symptoms suggestive of severe HFMD including continuous high fever, myoclonus, persistent vomiting, oral ulcers and vesicular erythema combined with high white blood cell count especially in young children. Prompt treatment is important because most cases died within one day after referral. The proportion of misdiagnosis at referral was considerable. Training courses on diagnosis and treatment of HFMD for clinicians should be provided widely and focused on diagnosis and classification of HFMD in order to reduce the case fatality.

## Competing interests

The authors declare that they have no competing interests.

## Authors’ contributions

NTBN, HVP and NNTM participated in the design of the study, performed data analysis, interpretation and wrote the manuscript. CQH, LNV and TMN and HCP were responsible for the collection and entry of data. LTN and LTP contributed to analysis and interpretation of data. All authors read and approved the final manuscript.

## Pre-publication history

The pre-publication history for this paper can be accessed here:

http://www.biomedcentral.com/1471-2334/14/341/prepub

## Supplementary Material

Additional file 1 Questionnaire. Click here for file
