# Supplementary material for: Epidemiological and clinical characteristics of children who died from hand, foot and mouth disease in Vietnam, 2011
Source: BMC Infect Dis. 2014 Jun 18;14:341. doi: 10.1186/1471-2334-14-341 (PMC4068316; doi:10.1186/1471-2334-14-341)
Supplement: Additional file 1 — Questionnaire. [file 1471-2334-14-341-S1.doc]

**Additional file 1:**

**QUESTIONNAIRE**

**(Using for Epidemiological and Clinical Characteristics of Children Who Died from Hand, Food and Mouth Disease in Vietnam, 2011)**

**1. GENERAL INFORMATION:**

1.1. Patient’s name:. . . . . . . . . . . . . . . . . . . . . . 1.2. Sex: Male  Female : 

1.3. Date of birth: . . . /. . . / . . . (...... months) 1.4. Ethnic: Kinh ; Ethnic minority  . . . . . . . .

1.5. Height: . . . . . . . . . . (cm) 1.6. Weight: . . . . . . (kg)

1.7. Parent’s name: . . . . . . . . . . . . . . . . . . . . . 1.8. Tel: . . . . . . . . .

1.9. Address: a. House number: . . . . . . . . . . . . . . . b. Street: . . . . . . . . . . . . . . . . . . . . . . . . . . . . . . . . . . . . .

c. Hamlet . . . . . . . . . . . . . . . . . . . . . d. Commune/Ward: . . . . . . . . . . . . . . . . . . . . . . . . . . . . .

e. District: . . . . . . . . . . . . . . . . f. Province : . . . . . . . . . . . . . . . . . . . . . . . . . . . . . .

1.10. School attendance/kindergarten:  At home  . If School attendance/kindergarten:

a. Name of school/kindergarten: . . . . . . . . . . . . . . . . . . . . . . . . . . . . . . . . . . . . . . . . . . . . . . . . . . . . . . . . .

b. Address: . . . . . . . . . . . . . . . . . . . . . . . . . . . . . . . . . . . . . . . . . . . . . . . . . . . . . .

1.11. Total of children in the family:. . . . . . . . . . . . Birth order? . . . . . . . . . . . . . . . . . . . . . . . . . .

1.12. Date of admission : . . . . / . . . ./ . . . . . . 1.12. Date of death: . . . . / . . . ./ . . . . . .

**2. EPIDEMIOLOGY**

2.1. History of pregnancy: a. Full-term pregnancy  b. Premature pregnancy 

2.2. History of birth a. Natural childbirth  b. Intervention birth 

2.3. Medical history included (syntrophus, chronic) Yes:  No: .. If yes: . . . . . . . . . . .

. . . . . . . . . . . . . . . . . . . . . . . . . . . . . . . . . . . . . . . . . . . . . . . . . . . . . . . . . . . . . . . . . . . . . . . . . . . . . . . . . . . . . .

. . . . . . . . . . . . . . . . . . . . . . . . . . . . . . . . . . . . . . . . . . . . . . . . . . . . . . . . . . . . . . . . . . . . . . . . . . . . . . . . . . . . . .

2.4. Nutritional status: a. Normal  b. Underweight  c. Overweight 

2.5. History of contact with HFMD patients: Yes:  No: . If yes:

a. Patient’s name: . . . . . . . . . . . . . . . . . . . . . . . . . c. Contact time: . . . . / . . . . / . . . .

c. Contact place:. . . . . . . . . . . . . . . . . . . . . . . . . . . . . . . . . . . . . . . . . . . . . . . . . . . . . . . . . . . . . . . . . . . . . . . . .

2.6. History of patient to health facilities for other reasons Yes:  No: . If Yes:

a. Time: . . . . / . . . . / . . . . b. Name of health facilities : . . . . . . . . . . . . . . . . . . . . . . . . . . .. .

2.7. History of patient’s family to health facilities for other reasons Yes:  No: . If Yes:

a. Name: b. Relation with patient:

c. Time: . . . . / . . . . / . . . . d. Name of health facilities:. . . . . . . . . . . . . . . . . . . .

**3. MEDICAL HISTORY AT HOME**

3.1. Date of onset: . . . . / . . . ./ . . .

3.2. Main symptoms of onset at home:

a. Fever: Yes: . No: . Temperature? . . . . (^0^C).

b. Rash: Yes: . No: .

c. Vomitting : Yes: . No: . d. Diarrhea: Yes: . No: .

e. Oral ulcers: Yes: ; No: 

f. Blister rash: Yes . No: . If yes, position:

Hand ; Foot ; Mouth ; Others: . . . . . . . . . . . . . . . . . . . . . . . . . . . . . . . . . . . . . . . . . . . . . . . . . . . . . . .

f. Other symptoms: . . . . . . . . . . . . . . . . . . . . . . . . . . . . . . . . . . . . . . . . . . . . . . . . . . . . . . . . . . . . . . . . . . . . . .

. . . . . . . . . . . . . . . . . . . . . . . . . . . . . . . . . . . . . . . . . . . . . . . . . . . . . . . . . . . . . . . . . . . . . . . . . . . . . . . . . . . . . .

3.3. Treatment before admission:

a. Buy medicine at pharmacy store:  b. Private health facilities: c. Commune heatlh center : 

3.4. Medicine used:

Pyrazolone: Yes:  No: . Glucocorticoid: Yes:  No: . Others: . . . . . . . . . . . . . . . . . . .

. . . . . . . . . . . . . . . . . . . . . . . . . . . . . . . . . . . . . . . . . . . . . . . . . . . . . . . . . . . . . . . . . . . . . . . . . . . . . . . . . . . . . .

**4. Clinical at the first health facility** *(skip this part if patient directly came to central level)*

4.1. Date of onset: . . . . .hour . . . . ; . . . . / . . . ./ . . .

4.2.. Name of the fist health facility (Commune health center/ hospital): . . . . . . . . . . . . . . . . . . . . . . . . . . . . . .

Reasons of admission: . . . . . . . . . . . . . . . . . . . . . . . . . . . . . . . . . . . . . . . . . . . . . . . . . . . . . . . . . . . . . . . . . . .

4.3. Admission diagnosis: a. HFMD . Class : 1 ; 2a ; 2b , 3 or 4 

b. Other disease:  (ghi rõ). . . . . . . . . . . . . . . . . . . . . . . . . . . . . . . . . . . . . . . . . . . . .

4.4..Referal diagnosis: a. HFMD . Class : 1 ; 2a ; 2b , 3 or 4 

b. Other disease:  (ghi rõ) . . . . . . . . . . . . . . . . . . . . . . . . . . . . . . . . . . . . . . . . . . . . . . . . . . . . . . . . . . . . . . . .

4.5. Main symptoms at admission:

a. Pulse: . . . . . /min; Temperature: . . . . (^0^C) Arterial pressure: . . . . . mm/Hg

b. Rash: Yes: . No: .

c. Vomitting : Yes: . No: . d. Diarrhea: Yes: . No: .

e. Oral ulcers: Yes: ; No: 

f. Blister rash: Yes . No: . If yes, position:

Hand ; Foot ; Mouth ; Others: . . . . . . . . . . . . . . . . . . . . . . . . . . . . . . . . . . . . . . . . . . . . . . . . . . . . . . .

g. Startling: Yes: ; No: 

h. Other symptoms: . . . . . . . . . . . . . . . . . . . . . . . . . . . . . . . . . . . . . . . . . . . . . . . . . . . . . . . . . . . . . . . . . . . . . .

. . . . . . . . . . . . . . . . . . . . . . . . . . . . . . . . . . . . . . . . . . . . . . . . . . . . . . . . . . . . . . . . . . . . . . . . . . . . . . . . . . . . . .

4.6. Treatment medicine : . . . . . . . . . . . . . . . . . . . . . . . . . . . . . . . . . . . . . . . . . . . . . . . . . . . . . . . . . . . . . . . . .

4.7. Date of referal: . . . . .hour . . . . ; . . . . / . . . ./ . . .

**5. Clinical at hospital:**

5.1. Hospital: . . . . . . . . . . . . . . . . . . . . . . . . . . . 5.2. date of admission: . . . .hour . . . . ; . . . . / . . . ./ . . .

Reasons of admission: referal  / self-coming 

5.3. Admission diagnosis: HFMD . Class : 1 ; 2a ; 2b , 3 hoặc 4 

Others:  . . . . . . . . . . . . . . . . . . . . . . . . . . . . . . . . . . . . . . . . . . . . . . . . . . .

5. 4. Diagnosis at death HFMD:  Others: . . . . . . . . . . . . . . . . . . . . . . . . . . . . . . . . . . .

5.5. Main symptoms at admission:

a. Pulse: . . . . . /min; Temperature: . . . . (^0^C) Arterial pressure: . . . . . mm/Hg

b. Rash: Yes: . No: .

c. Vomitting : Yes: . No: . d. Diarrhea: Yes: . No: .

e. Oral ulcers: Yes: ; No: 

f. Blister rash: Yes . No: . If yes, position:

Hand ; Foot ; Mouth ; Others: . . . . . . . . . . . . . . . . . . . . . . . . . . . . . . . . . . . . . . . . . . . . . . . . . . . . . . .

g. Startling: Yes: ; No: 

5.6. Clinical progress by day:

| **Symptom** | **D1** | **D2** | **D3** | **D4** | **D5** | **D6** | **D7** |
| --- | --- | --- | --- | --- | --- | --- | --- |
| 5.6.1. Fever (^0^C) |  |  |  |  |  |  |  |
| 5.6.2. Pulse (times/ min) |  |  |  |  |  |  |  |
| 5.6.3. Arterial pressure (mmHg) |  |  |  |  |  |  |  |
| 5.6.4. Sore throat |  |  |  |  |  |  |  |
| 5.6.5. Diarrhea |  |  |  |  |  |  |  |
| 5.6.6. Vomitting |  |  |  |  |  |  |  |
| 5.6.7. Mouth ulcer |  |  |  |  |  |  |  |
| 5.6.8. Blister rash |  |  |  |  |  |  |  |
| 5.6.9. Distressed |  |  |  |  |  |  |  |
| 5.6.10. Trembling limbs |  |  |  |  |  |  |  |
| 5.6.11. Stargging |  |  |  |  |  |  |  |
| 5.6.12. Eyes rolled |  |  |  |  |  |  |  |
| 5.6.12. Disturbances of consciousness  (Glasgow =......points) |  |  |  |  |  |  |  |
| 5.6.13. Coma |  |  |  |  |  |  |  |
| 5.6.14. Limb weakness |  |  |  |  |  |  |  |
| 5.6.15. Paralyzed cranial nerves |  |  |  |  |  |  |  |
| 5.6.16. Convulsions |  |  |  |  |  |  |  |
| 5.6.17. Sweating |  |  |  |  |  |  |  |
| 5.6.18. Veins raised on the skin |  |  |  |  |  |  |  |
| 5.6.19. Rapid pulse >150/min |  |  |  |  |  |  |  |
| 5.6.20. Hypotension |  |  |  |  |  |  |  |
| 5.6.21. Breathing fast |  |  |  |  |  |  |  |
| 5.6.22. Dyspnea |  |  |  |  |  |  |  |
| Others |  |  |  |  |  |  |  |

**6. LABORATORY**

**6.1. Tests detecting virus:**

- Feces: Date of taking sample: . . . . . ./ . . . /. . . . . .

Lab technique: Testing agency : . . . . . . . . . . . . . . . . . . . . Result: . . . . . . . . . . . . . . . .

- Throat swabs: Date of taking sample: . . . . . ./ . . . /. . . . . .

Lab technique: Testing agency : . . . . . . . . . . . . . . . . . . . . . Result: . . . . . . . . . . . . . . . .

- Vecicles fluid: Date of taking sample: . . . . . ./ . . . /. . . . . .

Lab technique: Testing agency : . . . . . . . . . . . . . . . . . . . . . Result: . . . . . . . . . . . . . . . .

**6.2. Other tests:**

| **Tests** | **D1** | **D2** | **D3** | **D4** | **D5** | **D6** | **D7** |
| --- | --- | --- | --- | --- | --- | --- | --- |
| 6.2.1. Red blood cells |  |  |  |  |  |  |  |
| 6.2.2. White blood cells |  |  |  |  |  |  |  |
| 6.2.3. Platelet |  |  |  |  |  |  |  |
| 6.2.4. Sedimentator |  |  |  |  |  |  |  |
| 6.2.5. Glucose Blood |  |  |  |  |  |  |  |
| 6.2.6. Troponine I |  |  |  |  |  |  |  |
| 6.2.7. CK (CPK) |  |  |  |  |  |  |  |
| 6.2.8. Ure |  |  |  |  |  |  |  |
| 6.2.9. Creatinine |  |  |  |  |  |  |  |
| 6.2.10. Na+ |  |  |  |  |  |  |  |
| 6.2.11. K+ |  |  |  |  |  |  |  |
| 6.2.12. Cl- |  |  |  |  |  |  |  |
| 6.2.13. AST |  |  |  |  |  |  |  |
| 6.2.14. ALT |  |  |  |  |  |  |  |
| 6.2.15. Protein DNT |  |  |  |  |  |  |  |
| 6.2.16. Glucose DNT |  |  |  |  |  |  |  |
| 6.2.17. Salt DNT |  |  |  |  |  |  |  |
| 6.2.18. Cells DNT |  |  |  |  |  |  |  |
| 6.2.19. Blood air |  |  |  |  |  |  |  |

**7. TREATMENT:**

**7.1. Follow-up:**

Decentralized follow-up (appropriate regimen) Yes: . No: .

Means for follow-up (appropriate regimen) Yes: . No: .

Treatment department: . . . . . . . . . . . . . . . . . . . . . . . . . . . . . . . . . . . . . . . . . . . . . . . . . . . . . . . . . . . . . . . . . . .

**7.2. Immunoglobulin treatment:**

7.2.1. Indication: Yes: . No: . If yes: Indication time : . . . hour . . . . . . . /. . . . /. . . . . .

Dose : . . . . . . . . . . . . . . . . . . . . . . . . Treatment begining time : . . . hour. . . . . . . /. . . . /. . . . . .

7.2.2. Respond after treatment:

7.2.2.1. Symptom improved

Pulse reduced : Yes: . No: . Time after using: . . . . . . . . . . . . . . . . . . . . . . . . . . . . . . . . . .

Fever reduced: Yes: . No: . Time after using: . . . . . . . . . . . . . . . . . . . . . . . . . . . . . . . . . .

Blood pressure turned normal: Yes: . No: . Time after using: . . . . . . . . . . . . . . . . . . . . . . .

Breathing turned normal : Yes: . No: . Time after using: . . . . . . . . . . . . . . . . . . . . . . .

Other symptoms : . . . . . . . . . . . . . . . . . . . . . . . . . . . . . . . . . . . . . . . . . . . . . . . . . . . . . . . . . . . . . . . . .

7.2.2.2. New/continued symptoms:

Pulse rise: Yes: . No: . Time after using : . . . . . . . . . . . . . . . . . . . . . . . . . . . . . . . . . .

Fever rise: Yes: . No: . Time after using : . . . . . . . . . . . . . . . . . . . . . . . . . . . . . . . . . . . . . . . .

Blood pressure swing: Yes: . No: . Time after using : . . . . . . . . . . . . . . . . . . . . . . . . . . . .

Breathing disorders: Yes: . No: . Time after using : . . . . . . . . . . . . . . . . . . . . . . . . . . . . . . . . . .

Other: . . . . . . . . . . . . . . . . . . . . . . . . . . . . . . . . . . . . . . . . . . . . . . . . . . . . . . . . . . . . . . . . . . . . . . . . . . .

**7.3. Continuously dialyze**

Indication : Yes: . No: . Indication time: h / /

Dialyze time : h / /

**7.3. Intensive care**

**7.3.1. Respiratory care:**

**7.3.1.1. Respiratory failure/no pulmonary edema**

a. Therapy of oxygen:

Indication : Yes: . No: . Time from indication to implement: . . . . . . . . . . . . . . . . . .

b. CPAP breathing:

Indication: Yes: . No: . Time from indication to implement : . . . . . . . . . . . . . . . . . .

c. Endotracheal intubation / artificial ventilation:

Indication : Yes: . No: . If yes: Time from indication to implement: . . . . . . . . . . . . . . . . . . . . . . . .

Artificial ventilation’s regime : . . . . . . . . . . . . . . . . . . . . . . . . . . . . . . . . . . . . . . . . . . . . . . . . . . . . . . . . . . . . . . .

Proper artificial ventilation parameter: Yes: . No: .

Adjust artificial ventilation parameter/blood air: . . . . . . . . . . . . . . . . . . . . . . . . . . . . . . . . . . . . . . . . . . .

Artificial ventilation tranquillizer:

Category : . . . . . . . . . . . . . . . . . . . . . . . . . . . . . . . . . . . . . . . . . . . . . . . . . . . . . . . . . . . . . . . . . . . . . . . .

Dose : . . . . . . . . . . . . . . . . . . . . . . . . . . . . . . . . . . . . . . . . . . . . . . . . . . . . . . . . . . . . . . . . . . . . . .

Effectiveness: Yes: . No: .

Test: Bood air

**7.3.1.2. Respiratory failure /Pulmonary edema:** Yes / No

Time from indication to care: . . . . . . . . . . . . . . . . . . . . . . . . . . . . . . . . . . . . . . . . . . . . . . . . . . . .

Care methods: . . . . . . . . . . . . . . . . . . . . . . . . . . . . . . . . . . . . . . . . . . . . . . . . . . . . . . . . . . . . . . .

Stop infusion: Yes: . No: .

Artificial ventilation:

Indication : Yes: . No: .

Time from indication to implement: . . . . . . . . . . . . . . . . . . . . . . . . . . . . . . . . . .

Artificial ventilation’s regime: . . . . . . . . . . . . . . . . . . . . . . . . . . . . . . . . . . . . . . . . . . . . . . . . . . .

Proper artificial ventilation parameter: Yes: . No: .

Artificial ventilation parameter/blood air : . . . . . . . . . . . . . . . . . . . . . . . . . . . . . . . . . . . . . . . . . .

Artificial ventilation tranquillizer :

Category :

Dose :

Effectiveness: Yes  No 

Dobutamin :

Indication: Yes  No 

Time from indication to implement: . . . . . . . . . . . . . . . . . . . . . . . . . . . . . . . . . . . . . . . .

Dose : . . . . . . . . . . . . . . . . . . . . . . . . . . . . . . . . . . . . . . . . . . . . . . . . . . . . . . . . . . . . . . . .

Proper dose adjustment : Yes  No 

Mirilnone :

Indication: Yes  No 

Time from indication to use : ………….

Dose:………………………………………….

Proper dose adjustment: Yes  No 

Furosemide :

Indication: Yes  No 

Time from indication to use:………….

Dose:………………………………………….

Proper dose adjustment: Yes  No 

Follow-up:

Blood air:………………………………..

X-ray:…………………………………

CVP:

Indication: Yes  No 

Time from indication to implement : Yes  No 

Adjust the speed basing on CPV results and clinical response: Yes  No 

**7.3.2. Blood circulation:**

**7.3.2.1. Follow-up:**

Follow-up method: continuously monitor , interupted : Every ……hour

Time from symptoms started to continuously monitor:………………………………………

Pulse follow-up: Yes: . No: .; Proper follow-up time: Yes: . No: .

Timing of appearance of changing pulse > 150/min to care…………

Timing of appearance of changing pulse >170 / min to care ………...

Blood pressure follow-up: Yes/ No ; proper follow-up time: Yes . No .

Hypertension: Yes  No 

Time from hypertension until treatment : . . . . . . . . . . . . . . . . . . . . . . . . . . . . . . . . . . . . . . . . . .

**7.3.2.2. Laboratory**

CKMB: Indication : Yes  No 

Time from indication to implement: . . . . . . . . . . . . . . . . . . . . . . . . . . . . . . . . . . . . . . . . . . . . . .

Troponin I: Indication: Yes  No 

Time from indication to implement : . . . . . . . . . . . . . . . . . . . . . . . . . . . . . . . . . . .

Heart ultrasound scan: Indication: Yes  No 

Time from indication to implement: . . . . . . . . . . . . . . . . . . . . . . . . . . . . . . . . . . .

**7.3.2.3. Treatment:**

Functional disorder of aortic ventricle of heart (myocarditis)

Dobutamin

Indication: Yes  No 

Time from indication to implement: . . . . . . . . . . . . . . . . . . . . . . . . . . . . . . .

Dose and proper dose adjustment: Yes/ No

Respond follow-up: Yes  No 

Mirinone : Indication: Yes  No 

Time from indication to implement:....

Dose and proper dose adjustment: Yes/ No

Respond follow-up: Yes/ No

CVP: Indication: Yes  No 

Time from indication to implement:…

CVP indicator : . . . . . . . . . . . . . . . . . . . . . . . . . . . . . . . . . . . . . . . . . . . . . . . . . . . . . . . .

Shock:

CVP: Indication: Yes  No 

` Time from indication to implement:…

CVP indicator: . . . . . . . . . . . . . . . . . . . . . . . . . . . . . . . . . . . . . . . . . . . . . . . . . . .

Adjust profusion according to CVP: Yes  No 

Infusion: Yes / No

Indication: Yes  No 

Category:…………….

Proper dose: Yes / No

Adjust infusion according to CVP: Yes: . No: 

Dopamin: Yes / No

Indication: Yes  No 

Time from indication to implement:……….

Proper dose: Yes: . No: .

Proper adjustment: Yes: . No: .

Dobutamin :

Indication: Yes  No 

Time from indication to implement:……….

Proper dose: Yes/ No

Proper adjustment: Yes/No

Adrenalin:

Indication: Yes  No 

Time from indication to implement:……….

Proper dose: Yes/ No

Proper adjustment: Yes/ No

**7.3.3. Nerve**

**7.3.3.1. Treat the cerebral edema:**

Indication: Yes  No 

Time from indication to implement:…………….

Methods:

Sit the patient up 30^0^: Yes  No 

Therapy of oxygen by mask: Indication: Yes  No 

Endotracheal intubation

Indication: Yes  No 

Time from indication to implement:…………………………………….

Artificial ventilation:

Indication: Yes  No 

Time from indication to implement:………….

Artificial ventilation’s regime:…………………

Proper artificial ventilation parameter: Yes/ No:………………………

Adjust artificial ventilation parameter/ blood air

Artificial ventilation tranquillized:

Category:

Dose:

Effectiveness: Yes: . No: 

Treat seizures: Yes  No 

Infusion dose according to demand: Yes  No 

**7.3.3.2. Breathing rate disorder:**

Indicate the mechanical artificial ventilation: Yes/ No

Time from indication to implement: ………………………….

Artificial ventilation method, parameter, blood air

Seizures:

Seizures care: Yes  No 

Medicine:

Dose:………………………..

Effectiveness: Yes . No .

**7.3.3. Adjust Electrolysis**

Ions tests:

Electrolysis disorder: Yes  No . If yes:

Time from indication to implement :

Proper adjustment method: Yes/ No

Blood glucose : Disorder : Yes: . No: .

Treatment: . . . . . . . . . . . . . . . . . . . . . . . . . . . . . . . . . . . . . . . . . . . . . . . . . . . . .

**8. OTHER INFORMATION**

. . . . . . . . . . . . . . . . . . . . . . . . . . . . . . . . . . . . . . . . . . . . . . . . . . . . . . . . . . . . . . . . . . . . . . . . . . . . . . . . . . . . . .

. . . . . . . . . . . . . . . . . . . . . . . . . . . . . . . . . . . . . . . . . . . . . . . . . . . . . . . . . . . . . . . . . . . . . . . . . . . . . . . . . . . . . .

. . . . . . . . . . . . . . . . . . . . . . . . . . . . . . . . . . . . . . . . . . . . . . . . . . . . . . . . . . . . . . . . . . . . . . . . . . . . . . . . . . . . . .

. . . . . . . . . . . . . . . . . . . . . . . . . . . . . . . . . . . . . . . . . . . . . . . . . . . . . . . . . . . . . . . . . . . . . . . . . . . . . . . . . . . . . .

. . . . . . . . . . . . . . . . . . . . . . . . . . . . . . . . . . . . . . . . . . . . . . . . . . . . . . . . . . . . . . . . . . . . . . . . . . . . . . . . . . . . . .

. . . . . . . . . . . . . . . . . . . . . . . . . . . . . . . . . . . . . . . . . . . . . . . . . . . . . . . . . . . . . . . . . . . . . . . . . . . . . . . . . . . . . .

. . . . . . . . . . . . . . . . . . . . . . . . . . . . . . . . . . . . . . . . . . . . . . . . . . . . . . . . . . . . . . . . . . . . . . . . . . . . . . . . . . . . . .

. . . . . . . . . . . . . . . . . . . . . . . . . . . . . . . . . . . . . . . . . . . . . . . . . . . . . . . . . . . . . . . . . . . . . . . . . . . . . . . . . . . . . .

Người điều tra
